# Resilience among Tunisian women: Exploring the impact of coping Strategies and sociodemographic influences

**DOI:** 10.1192/j.eurpsy.2025.1616

**Published:** 2025-08-26

**Authors:** H. Mhiri, I. Chaari, I. Mannoubi, N. Boussaid, F. Charfeddine, L. Aribi, N. Messedi, J. Aloulou

**Affiliations:** 1 Psychiatric department “B”, Hedi Chaker University Hospital, Sfax, Tunisia

## Abstract

**Introduction:**

Tunisian women, seen as symbols of freedom, have achieved social equality despite various challenges. This study examines the role of sociodemographic factors and coping strategies in shaping psychological resilience among Tunisian women.

**Objectives:**

To investigate how sociodemographic factors and coping styles influence resilience in Tunisian women.

**Methods:**

This cross-sectional study targeted Tunisian women aged 18 and above through an online survey between June and August 2024. Sociodemographic data were collected, coping styles were measured using the Brief COPE inventory, and resilience was assessed via the 25-item Connor-Davidson Resilience Scale (CD-RISC 25).

**Results:**

Data from 695 women (mean age = 36.72 ± 12.23) showed that 90.9% had university education and 34.1% were unemployed. Personal income was the main income source for 61.7%. The mean resilience score was 68.26 ± 14.09, with 26.3% exhibiting low resilience. Higher resilience was significantly associated with university education (p < 0.001) and higher economic status; 34.3% of those in lower brackets showed low resilience (p = 0.007). Personal income also predicted resilience (p < 0.001). Regarding coping, resilience positively correlated with problem-focused (r = 0.496, p < 0.001) and emotion-focused coping (r = 0.271, p < 0.001), but was negatively associated with avoidance coping (r = -0.093, p < 0.05).

**Conclusions:**

This study reveals that resilience among Tunisian women is linked to education, economic status, and income, with effective coping strategies (problem- and emotion-focused) enhancing resilience. Findings underscore the importance of economic empowerment and effective coping skills in strengthening resilience among women.

**Disclosure of Interest:**

None Declared

